# Importance of Attachment Efficiency in Determining the Fate of PS and PVC Nanoplastic Heteroaggregation with Natural Colloids Using a Multimedia Model

**DOI:** 10.1021/acs.est.4c10918

**Published:** 2025-03-03

**Authors:** Fazel Abdolahpur Monikh, Joris T. K. Quik, Mark R. Wiesner, Andrea Tapparo, Paolo Pastore, Hans-Peter Grossart, Jarkko Akkanen, Raine Kortet, Jussi V.K. Kukkonen

**Affiliations:** † Department of Chemical Sciences, University of Padua, Via Francesco Marzolo, 1, 35122 Padua, Italy; ‡ Department of Environmental and Biological Sciences, University of Eastern Finland, 80101 Joensuu, Finland; § Institute for Nanomaterials, Advanced Technologies, and Innovation, Technical University of Liberec Bendlova 1409/7, 460 01 Liberec, Czech Republic; ∥ National Institute for Public Health and Environment (RIVM), Centre for Sustainability, Health and Environment, Antonie van Leeuwenhoeklaan 9, 3721 MA Bilthoven, The Netherlands; ⊥ Department of Civil and Environmental Engineering, Duke University, Durham, North Carolina 27708, United States; # Center for the Environmental Implications of NanoTechnology (CEINT), Duke University, Durham, North Carolina 27708, United States; ¶ Department of Plankton and Microbial Ecology, Leibniz Institute for Freshwater Ecology and Inland Fisheries, Stechlin, 16775 Berlin, Germany; ∇ Institute of Biochemistry and Biology, Potsdam University, 14469 Potsdam, Germany; ○ Department of Environmental and Biological Sciences, University of Eastern Finland, Kuopio 70211, Finland

**Keywords:** SimpleBox4Plastics model, plastic fate, freshwater, natural organic matter, microplastics

## Abstract

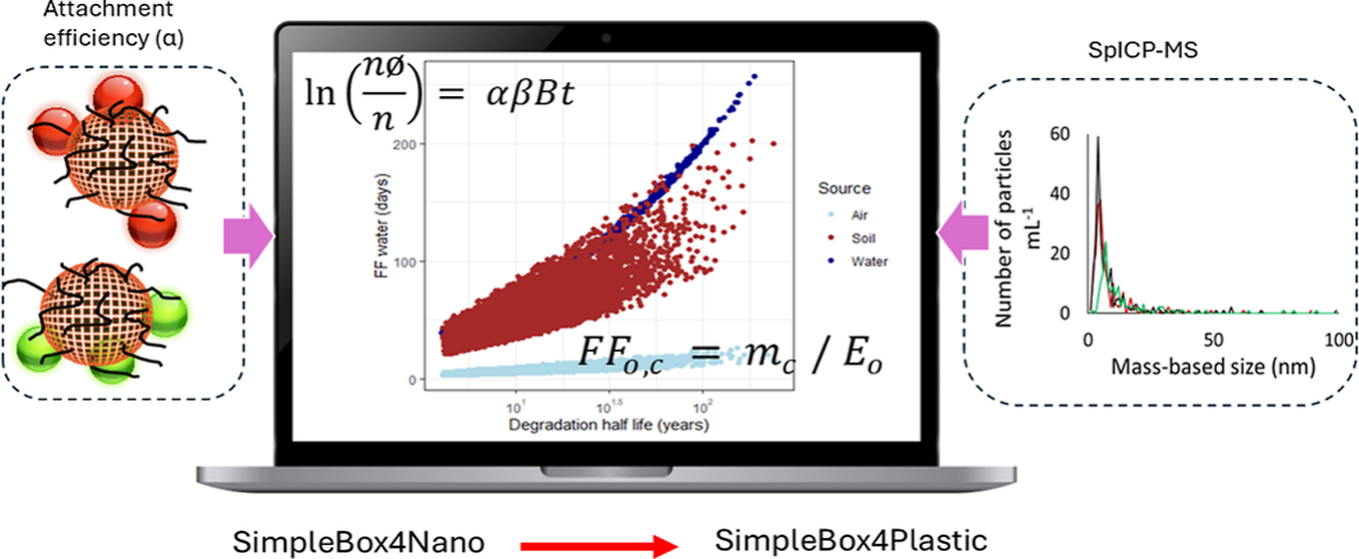

Here, we assessed the heteroaggregation of polystyrene (PS) and poly(vinyl chloride) (PVC) nanoplastics with SiO_2_ as a model of natural colloids. Homoaggregation and heteroaggregation were evaluated as a function of CaCl_2_ (0–100 mM) and natural organic matter (NOM) (50 mg L^–1^) at a designated concentration of nanoplastics (200 μg L^–1^). Critical coagulation concentrations (CCC) of nanoplastics were determined in homoaggregation and heteroaggregation experiments with SiO_2_ and CaCl_2_. The attachment efficiency (α) was calculated by quantifying the number of nanoplastics in the presence of CaCl_2_, NOM, and SiO_2_ using single-particle inductively coupled plasma mass spectrometry (spICP-MS) and pseudo-first-order kinetics. The calculated α was fed into the SimpleBox4Plastics model to predict the fate of nanoplastics across air, water, soil, and sediment compartments. Nanoplastics exhibited high stability against homoaggregation, while significant heteroaggregation with SiO_2_ occurred at CaCl_2_ concentrations above 100 mM. The influence of NOM was also evaluated, showing a reduction in heteroaggregation with SiO_2_ for both nanoplastic types. Sensitivity analysis indicated that the degradation half-life of the tested nanoplastics had a more significant impact on persistence than did α. The results emphasize the environmental stability of nanoplastics, particularly in freshwater and soil compartments, and the critical role of NOM and emission pathways in determining their fate.

## Abstract 2

This research examines the aggregation behavior of polystyrene and polyvinyl chloride nanoplastics with natural colloids, highlighting how environmental factors like calcium ions and natural organic matter influence their stability and persistence in ecosystems.

## Introduction

Nanoplastics (here particles with size <1 μm)^1^ are formed mostly upon the degradation of plastic waste in the environment. Upon entering aquatic systems, nanoplastics may undergo alterations in their form and chemistry. These changes can include aggregation with various particulate materials in water and transformation processes such as photochemical transformation and interaction with natural organic matter (NOM), which critically influence the transport, fate, and toxicity of nanoplastics in the aquatic environment.^2^ Like other nanoparticles,^3^ nanoplastics may exhibit distinct behavior in achieving equilibrium partitioning compared to dissolved chemicals. Consequently, standard tests employed for determining partitioning or adsorption coefficients, such as *K*_ow_ and *K*_d_, necessitate special consideration due to the potential deviation from thermodynamic expectations over varying time scales.^4^

Nanoplastics have the potential to collide with and adhere to particles of the same type, known as homoaggregation, or to particles of different types, termed heteroaggregation.^5,6^ Within environmental contexts, the concentrations of nanoplastics are markedly lower than those of naturally occurring colloids such as iron oxides, silicon dioxide (SiO_2_), clay minerals, etc., considering both mass and numerical aspects.^7^ This increases the likelihood of nanoplastic heteroaggregation over homoaggregation in real environments. Although an increasing number of studies have been conducted in recent years to understand the heteroaggregation of nanoplastics with natural colloids,^8−10^ the influence of different physicochemical parameters of nanoplastics—such as particle size and chemical composition—and environmental factors (e.g., pH, ionic strength, and NOM)^8,11^ on heteroaggregation remains largely unknown. Considering the limitless number of parameter combinations that might influence the heteroaggregation of nanoplastics, it is not feasible to investigate each condition experimentally. This highlights the importance of modeling to understand the fate of heteroaggregation and subsequently of nanoplastics in the environment.

Several models have been developed to investigate the behavior of nanoparticles in the environment. Powerful tools suited for this purpose are environmental fate models such as MendNano^12^ and NanoDUFLOW.^13^ These models can be used to predict exposure levels and transport behavior of nanoparticles and to support a proactive risk assessment. Another model is SimpleBox4Nano (SB4N),^13^ which utilizes first-order kinetics to estimate environmental background concentrations for colloids in an environmental system composed of air, soil, water, and sediment compartments represented as boxes.^13^ SB4N was adapted to SimpleBox4Plastic (SB4P), which operates on three scales: regional, continental, and global.^14^ Particles within the model can exist in each compartment in three forms: freely dispersed, heteroaggregated with natural colloidal particles (<450 nm), and attached to larger particles (>450 nm).^13^ The required input for SB4P can be categorized as follows: scenario settings, substance properties (e.g., attachment efficiency (α), dissolution and degradation rate constants), and emission rates. However, determining the α of nanoplastics to other colloids, which is a pivotal aspect in comprehending the characteristics of nanoplastics and their interactions within a specific system, remains challenging. In colloidal science, α is a dimensionless parameter that expresses the ratio of successful particle collisions to the total number of collisions^15^ and is highlighted as a critical property for evaluating nanoparticles’ fate in the environment.

Quantifying heteroaggregation and isolating individual α values from experimental data is an approach to determining α that poses significant challenges due to the multitude of concurrent competing processes of particle–particle collision.^16^ Often, the methodology employed in investigating homoaggregation may not be readily adaptable to heteroaggregation, primarily due to the need to experimentally track target particles (such as engineered nanoparticles or nanoplastics) and the inherently greater complexity of the environmental system such as the presence of natural colloids and NOM which affects values of α.^17^ Consequently, research on heteroaggregation remains relatively limited in comparison. Attempts have been made to adapt methods used to investigate homoaggregation to heteroaggregation, by tracking the average size evolution of aggregating particles, e.g., using dynamic light scattering (DLS).^18^ However, this approach cannot differentiate particle types as needed in a study of heteroaggregation. A more general approach to quantify heteroaggregation involves measurements of individual target particles remaining in suspension over time after heteroaggregates are removed via settling^19^ or filtration.^3^ In the case of particles containing metals, measuring the mass or number of particles by using inductively coupled plasma mass spectrometry (ICP–MS) and single particle (sp)ICP–MS can provide information on heteroaggregation.^20,21^ However, these methods face limitations when dealing with nanoplastics as they cannot be detected by ICP–MS. Recent advancements involving the entrapment of rare elements in polymeric structures have enabled the tracking of nanoplastics in such intricate environments.^6^ For example, in our recent publications, we applied gadolinium (Gd) as a rare element inside polymeric particles to mimic nanoplastics and investigate the uptake of the particles by plants and their trophic transfer.^22^ A previous review paper has provided detailed information about the application of this methodology for nanoplastic fate assessment.^22^

The objective of the current study is to obtain and use α values in conjunction with SB4P to understand how the variation in the chemical composition, particularly density and hydrophobicity, of nanoplastics affects their retention in different environmental compartments with their specific environmental conditions. Accordingly, we first determined the α value for heteroaggregation of PS and PVC nanoplastics with SiO_2_, as a model of natural colloids, in the presence of NOM and different ionic strengths. To facilitate quantification of the nanoplastics, Gd ions are entrapped inside the nanoplastics, allowing the application of spICP-MS to measure the particle numbers over time in the experimental systems. The obtained data on the number of particles over time are used to determine α to subsequently be applied in SB4P.

## Methods and Materials

### Theoretical Context

#### Attachment Efficiency (α)

The theoretical context and method for α measurement using a batch test has been described in detail previously^19^ and is briefly summarized here. The settling rate of individual nanoplastics is negligible due to their low density and small particle size. Instead, nanoparticles collide with natural particles, potentially leading to the attachment of the two particles and the subsequent settling of the resulting in larger aggregates.

The values of α range from 0 to 1, where α = 1 indicates a high tendency for particles to attach to other particles or surfaces. The modified Smoluchowski equation describes the transport processes governing how nanoparticles move toward a surface and the probability of attachment following collisions between particles.^23^ This equation illustrates the rate of change in the particle number concentration, which represents particles that have not undergone heteroaggregation with colloids over time. Therefore, a logarithmic plot of the initial nanoparticle concentration divided by the concentration of nanoparticles that have not formed heteroaggregates with natural colloids over time (eq 1) is expected to show a pseudo-first-order reaction. The slope of this linear relationship is anticipated to be equivalent to that of αβ*B*. In the initial phases of heteroaggregation, the breakup of the aggregate can be considered insignificant.
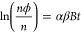
1where β is the second-order collision rate constant (volume time ^–1^ number ^–1^), *B* is the concentration of colloids (number volume ^–1^), *n* is the nanoparticle number concentration per unit of volume, and *n*ϕ is the initial nanoparticle number concentration per unit volume (i.e., the concentration at time *t* = 0). To determine the α_total_ for any given system, αβ*B* needs to be normalized by β*B* as slope αβ*Bt* represents α at a constant value of β*B*. Empirically, β*B* can be determined by modifying the conditions of the system to eliminate barriers to attachment, where α = 1 (referred to as favorable-attachment efficiency α_fav_ = 1), while keeping the dominant nanoparticle and colloid transport mechanisms unchanged and maintaining a constant colloid concentration.^23^ For instance, by increasing the ion strength of the system, using NaCl and CaCl_2_, it is possible to achieve α_fav_ = 1. The rate constant for an α_fav_ system determined from data plotted according to eq 1 is equal to β*B*. Subsequently, the α_total_ for the unadjusted system can be determined by normalizing the rate constant as shown in eq 2.
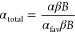
2

#### Critical Coagulation Concentration (CCC)

The aggregation-salt concentration plot typically demonstrates two discernible regimes: reaction-limited cluster aggregation (RLCR) and diffusion-limited cluster aggregation (DLCA) regimes. In the RLCR regime, aggregation occurs via a different mechanism. Instead of relying solely on diffusion, particles can also aggregate through specific chemical or physical interactions such as electrostatic attraction or van der Waals forces. In this regime, the rate of aggregation is determined by the frequency of encounters between particles and the likelihood that they stick together upon collision. In the DLCA regime, particles undergo random Brownian motion and aggregate when they collide and attach. This process is primarily driven by diffusion, where particles move in a fluid medium due to random motion. In this regime, the rate of aggregation is limited by diffusion of particles through the medium. The point of transition between these regimes, termed the CCC, serves as a pivotal indicator in understanding the underlying mechanisms governing nanoparticle aggregation dynamics.

#### Fate Factors

We utilized fate factors (FF) as an output variable, indicating the persistence of these materials in an environmental compartment and mitigating the uncertainty of emissions. These factors represent the residence time of the particles in a specific compartment. Since SB4P does not provide this directly as an output, it necessitates the calculation
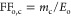
3where FF_o,c_ is the fate factor [days] for a particle in compartment c as emitted to compartment o, *m*_c_ is the output for steady-state mass [kg] for compartment c, and *E*_o_ [kg day^–1^] is the emission of a particle emitted to compartment o: air, soil, or freshwater. This is in line with the commonly applied approach in life cycle impact assessment modeling.^24,25^

## Materials

The materials and chemicals used in this study were purchased from Sigma-Aldrich unless otherwise specified. Spherical PS (∼250 nm) and PVC (∼250 nm) were designed by our group and were custom-synthesized by cd-bioparticles (NY 11967, USA) to our specifications. The company claimed that the Gd ions are entrapped inside the particles and not on the surface. To ensure the Gd ions are inside the particles as we requested and not released from the particles during the application, we have performed a comprehensive characterization^22^ (see next section). Water was deionized by reverse osmosis and further purified using a Millipore MQ system. The particles were stabilized with a Tween 20 (1%). The density of PS and PVC used in this study were 1.3–1.35 and 1.4–1.7 g cm^3^, respectively, as reported by the producer due to the presence of Gd in the particles. Silicon dioxide nanoparticles of 250–300 nm were used as a model of natural colloids, which was purchased from Sigma-Aldrich. SiO_2_ was purchased in powder form, and the particles were dispersed in water without any functionalization (uncovered).

### Particle Characterization

The hydrodynamic size of the particles and the zeta potential (ζ) were assessed by utilizing a Zetasizer Nanodevice (Malvern Panalytical, Malvern, UK). Particle shape was determined through transmission electron microscopy (TEM) using a JEOL JEM-2100F instrument operated at 200 kV. A scanning electron microscope (Zeiss Sigma HD|VP, Carl Zeiss NTS, Cambridge, UK)-EDX was used with 4 kV for elemental mapping of the nanoplastics. To assess hydrophobicity, a droplet of particle dispersion was dried on an aluminum surface, and the contact angle was measured using Milli-Q water at room temperature, employing a KSV Cam 200 contact angle instrument. The concentration of Gd ions and the number of particles were quantified using an ICP–MS instrument (PerkinElmer NexION 350D), operating in both single-particle and standard modes.

## Experimental Setup for Determining the α

### Measuring the CCC

First, we determined the CCC for the homoaggregation of the nanoplastics as well as their heteroaggregation with SiO_2_. The CCC of the particles was determined by measuring their size over different CaCl_2_ concentrations using DLS.^26^ To determine the CCC for the particles upon homoaggregation, 10 mg L^–1^ PS and PVC were mixed with CaCl_2_ concentrations of 10, 50, 100, and 1000 mM, separately. To determine the CCC for PS or PVC heteroaggregation with (10 mg L^–1^) SiO_2_, solutions of CaCl_2_ (0.1, 1, 10, 50, 100, 150, and 200 mM) were used. The selected concentrations were chosen based on the detection limit of the DLS instrument, which is approximately 10 mg L^–1^.^21^ Accordingly, the particles were introduced into a system containing the corresponding CaCl_2_, and their hydrodynamic sizes were promptly measured over 10 min. The obtained size was plotted over time to determine the CCC. Previous studies have shown that the fast interactions between particles and colloids are likely to occur within the first minutes.^3,23^ Therefore, the 10 min duration and interval were selected to be as short as possible to ensure prompt detection after sample preparation.

### Quantification of Nanoplastic Number in Different Systems

To determine α in a batch test, 200 μg L^–1^ nanoplastics (PS or PVC) were introduced into a system containing 20 mg of SiO_2_ and varying concentrations of CaCl_2_ (0.1, 1, 10, or 50 mM) in MQ water adjusted to pH 7.8 with 0.1 M NaOH. The samples were mixed by hand for 5 s in plastic tubes. The nanoplastic concentrations were chosen to establish the most environmentally relevant scenario feasible^27^ while detectable with a high sensitivity. The concentration of SiO_2_ was arbitrarily set to be 2 orders of magnitude higher than that of the nanoplastics. The α of nanoplastics onto SiO_2_ remains independent of these initial concentrations, which demonstrates that the number concentration of SiO_2_ (and its associated available surface area) is substantially higher than that of the nanoplastics.^3^ The selection of Ca^2+^ and their ranges was guided by OECD test number 318.^28^ Divalent ions like Ca^2+^ are commonly found at concentrations capable of influencing colloidal stability.^29^ To determine the influence of NOM on heteroaggregation of nanoplastics and SiO_2_, nanoplastics were introduced in a system containing 20 mg of SiO_2_, 10 mM CaCl_2_, and 50 mg L^–1^ dissolved NOM. The NOM was collected from the Hietajarvi S catchment area in Finland and dissolved according to the following method.^29^ This concentration of NOM was selected to represent the average to high concentrations of NOM in natural freshwater of the EU.^29^ A minimum of five replicate tests were conducted for each system. The setup of the spICP-MS is given in the Supporting Information (Table S1).

### Calculating Attachment Efficiency (α)

The measured number concentration of the nanoplastics was plotted against the aggregation time (minutes), generating a removal curve for the particles in each system. We calculated both *Ct*_0_/*Ct* and ln (*Ct*_0_/*Ct*) for the initial 5 data points, where *Ct*_0_ represents the particle count at time 0 and *Ct* represents the particle count at the specified time (*t*). The natural logarithm of the first five data points was plotted as a function of time to linearize the removal curves (pseudo-first-order kinetics).^23^ A fundamental assumption is that during the initial attachment phase (first five data points), the occurrence of breakup of the heteroaggregates is negligible. We calculated the slope of a regression line through the linear portion, which represents the quantity mM β*B* (eq 2) for nanoplastics with the SiO_2_ colloids. We selected these time points to be included in the linear regression because (1) the attachment period commences at the first measured time point, (2) it encompasses three or more consecutive points, and (3) it yields the highest coefficient of determination (*R*^2^).^23^ We determine the value of α for each system by using the obtained value of αβ*B* in eq 2, the known concentration of SiO_2_ colloids, and normalizing it by the slope for favored heteroaggregation at the CCC, where α is 1.

### Simple Box Model

In this study, we applied SimpleBox4Plastics (v4.04)^30^ as earlier described by Quik et al.,^14^ for the calculation of FF. To compare the effect of the calculated α and other variables related to the characteristics of the particles, these are applied probabilistically to determine their effect on the persistence of these particles in air, water, sediment, and soil. All input variables, not related to the characteristics of the particles, are taken deterministically using the default values already present in SB4P. The probabilistic calculations were performed using the @Risk Excel plugin (v8, Palisade) following the distribution given in the Supporting Information (Section 2). These include variations in size and density and uncertainty in the degradation and fragmentation rate constants in addition to the range in attachment efficiencies based on the measurements reported here. Both uniform and triangular distributions were used. A uniform distribution is used when it is expected that the values vary equally between a minimum and a maximum. A triangular distribution is used when values vary normally around a top value, i.e., the most likely value. This is preferred over normal distribution, to prevent extreme values from having a disproportionate impact. Furthermore, 10,000 iterations were performed. The air, freshwater + sediment, soil, and seawater + sediment compartments are considered for a scenario at the continental scale. Emissions are considered to be in air, freshwater, and soil. The subcompartments from soil (e.g., agricultural, natural, other soil) and freshwater (e.g., lake and river) are not separately considered to produce concise results. This means that three emission routes lead to different environmental compartments resulting in a total of 17 FF (see Table S2). These FFs are not just one value, considering the 10,000 possible interactions. Further details on model parameterization are reported in the Supporting Information (Section 2).

### Data Analysis

Here we used @Risk to probabilistically run the latest SB4P model according to the method reported previously.^14^ The graphs were plotted using Microsoft Office 2022 and R (v.4.3.1) (https://www.R-project.org/). Statistical evaluation of the data for normality was conducted utilizing a Shapiro–Wilk test through IBM SPSS statistics 29.0.2.

## Results and Discussion

### Particle Characterization

**Figure 1:**
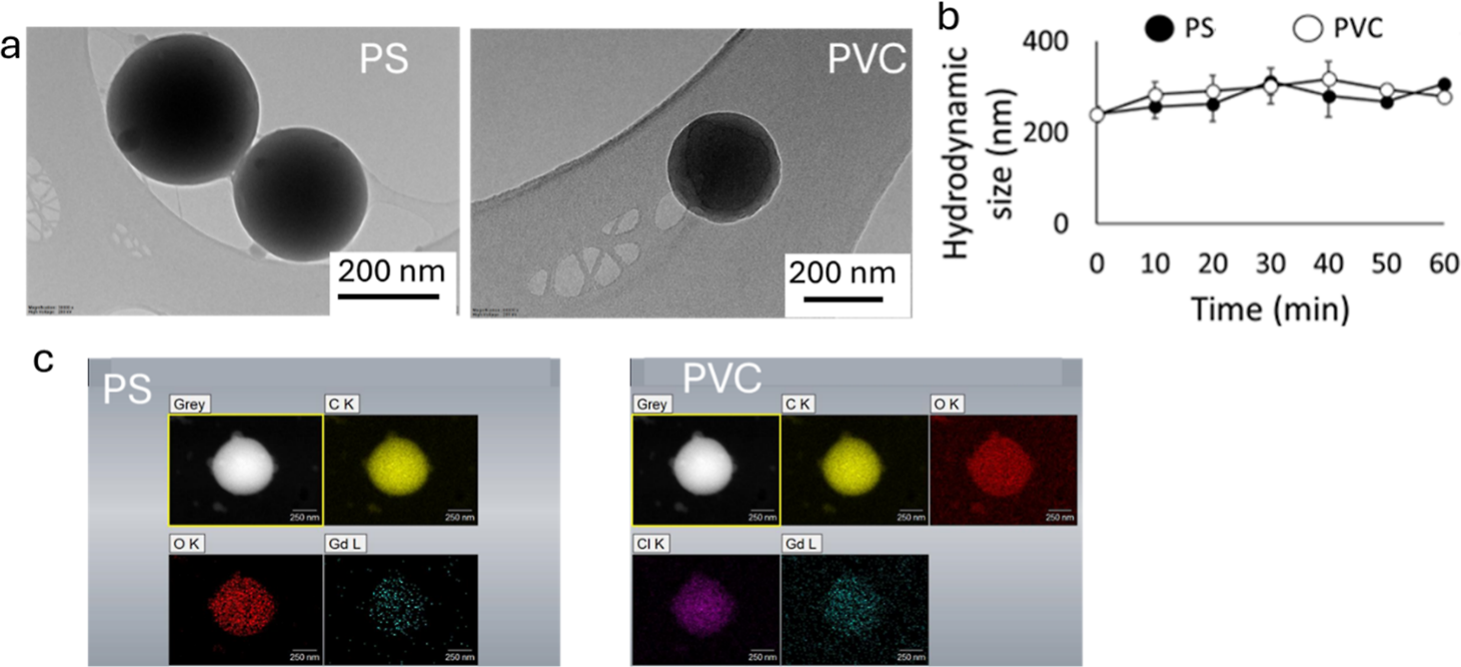
(a) TEM image of PS nanoplastics and PVC nanoplastics. (b) The hydrodynamic size (nm) of the particles was measured over 1 h using DLS to show the homoagglomeration profile of the particles in MQ water. (c) SEM-EDS elemental mapping of PS (the particles, carbon, oxygen, and Gd) and PVC (the particles, carbon, oxygen, chlorine, and Gd) nanoplastics showing the distribution of Gd in the particles.

The spherical pristine PS and PVC nanoplastics were thoroughly characterized by using DLS and spICP-MS (Figure 1). The nominal particle diameters for PS and PVC were both 250 nm, with polydispersity indices (PDI) of 0.1 and 0.2, respectively. Analysis using DLS revealed hydrodynamic diameters consistent with nominal sizes, corroborated by TEM observations based on the average size of 100 particles per nanoplastic type, indicating a uniform size distribution (Figure 1a,b) for the nanoplastics and SiO_2_. The TEM image further confirmed that the SiO_2_ particles (Figure S1) used in this study exhibited a spherical morphology with an approximate size of 250 nm, matching the dimensions of the nanoplastics. PS nanoplastics exhibited a contact angle of 80 ± 2°, signifying higher hydrophobicity compared to PVC nanoplastics with a contact angle of 60 ± 1°. Furthermore, ζ measurements conducted in Milli-Q water indicated a negative charge for PS (−46 ± 2 mV), PVC (−48 ± 1 mV), and SiO_2_ (−27 ± 4 mV) particles. This negative ζ suggests a propensity for particle–particle repulsion, minimizing agglomeration tendencies.^27^

### Particle Stability

To perform an aggregation-related study using nanoparticles, it is important to ensure the stability of the pristine particles against aggregation and transformation to prevent uncontrolled changes in the particles.^29^ Investigation of nanoplastics’ stability in the context of homoaggregation involved monitoring hydrodynamic sizes using DLS over a 1 h duration (Figure 1b). The size of the particles did not increase significantly over time, indicating stability against homoaggregation. To ensure that the detectable Gd quantity within the particles using spICP-MS is representative of the number of particles, we conducted a comparison between the number of nanoplastics measured by spICP-MS and both the nominal concentration and particle count measured by DLS. Our results revealed statistically insignificant differences in the number of nanoplastics measured by the different techniques for both particle types (Figure S2).

Scanning electron microscopy (SEM) images coupled with energy-dispersive spectroscopy (EDS) analysis depicted the elemental distribution of Gd within PS and PVC nanoplastics (Figure 1c). It is noteworthy that the Gd ions were entrapped within the particles rather than being present on their surface. The Gd content within the particles was quantified to be 9.7%–11%.^22^ To ensure the retention of Gd within the particles and prevent leaching, we initially assessed particle stability by examining Gd ions in the nanoplastics dispersion in MQ water following a 72 h mixing at room temperature using spICP-MS. Importantly, no Gd ions were detectable in the supernatants, indicating robust particle stability and minimal leaching.

### Homo- and Heteroaggregation

First, we determined the CCC values of PS and PVC nanoplastics in different CaCl_2_ concentrations using DLS. The CCC values for both particles were not found even in a ∼1000 mM CaCl_2_ solution, indicating the stability of the particles against homoaggregation. The measured ζ values are reported in Table S3. Then, we measured the CCC for PS and PVC nanoplastics in combination with SiO_2_ particles (PS–SiO_2_ and PVC–SiO_2_) in a CaCl_2_ solution, which was ∼100 mM. At this CaCl_2_ concentration, the α is assumed to be 1 (favorable attachment, α = 1).^19^

**Figure 2:**
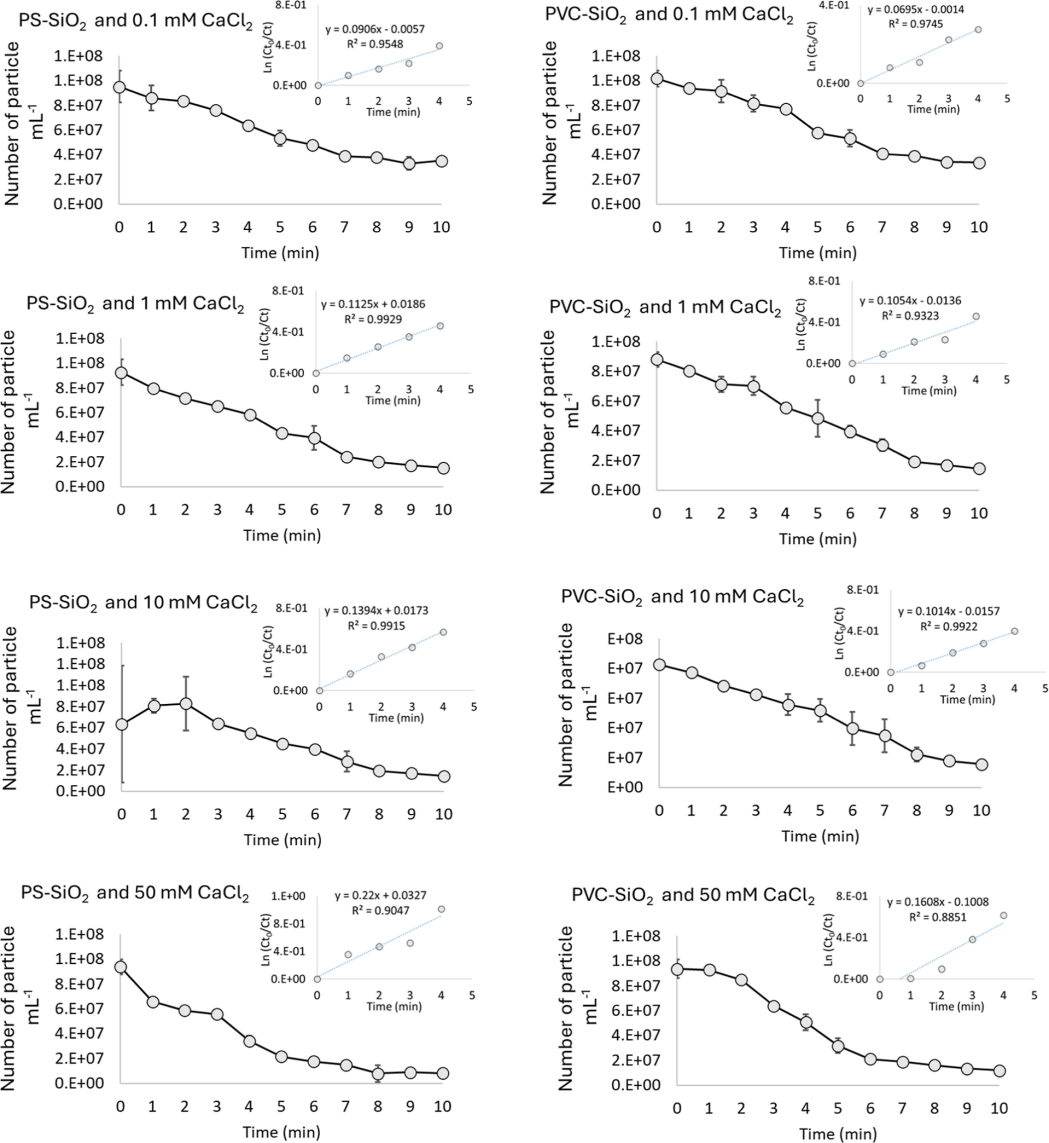
Number of PS and PVC nanoplastics in the presence of SiO_2_ and varying ionic strengths were measured using spICP-MS over 10 min. The results show the mean ± standard deviation. Calculated (*Ct*_0_/*Ct*) and the natural logarithm of (*Ct*_0_/*Ct*) for the first five data points are shown in the smaller graphs.

The number of nanoplastics (PS or PVC) in the medium with 20 mg L^–1^ SiO_2_ and different concentrations of CaCl_2_ was measured using spICP-MS for 10 min. As shown in Figure 2, the number of PS and PVC nanoplastics decreased in the supernatant over time, indicating the removal of particles from the dispersion phase. Considering that homoaggregation of PS–PS and PVC–PVC in the dispersion is not occurring due to the stability of the particles in this CaCl_2_ solution against homoaggregation, the only explanation for the decreased concentration of the nanoplastics is heteroaggregation of the particles with SiO_2_. A previous study showed that the CCC of 2 mM L^–1^ for both CaCl_2_ and MgCl_2_ was reached for fragmental PET particles, suggesting that in freshwater, nanoplastics are likely to aggregate rapidly under more alkaline conditions.^31^ The highest aggregation rate is expected in marine water due to the combination of high ionic strength (≈0.7 M L^–1^), a high concentration of multivalent ions (e.g., Ca ^2+^, Mg^2+^), and low NOM content. We determined (*Ct*_0_/*Ct*) and the natural logarithm of (*Ct*_0_/*Ct*) for the first five data points (Figure 2), where *Ct*_0_ is the number of particles at time 0 and *Ct* is the number of particles at the time of interest. The graph of the first five data points was plotted because the coefficient of determination (*R*^2^) was higher than 90%. Removal curves for all systems followed pseudo-first-order kinetics, allowing for linear regression, assuming that newly formed heteroaggregates did not break up.

**Figure 3:**
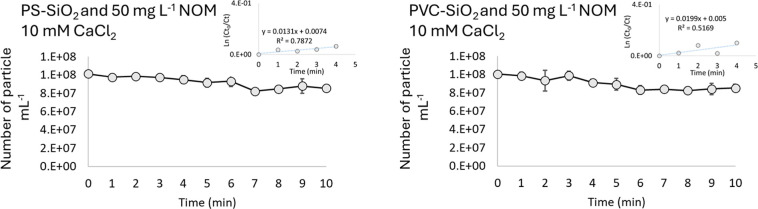
Number of PS and PVC nanoplastics in the presence of SiO_2_, 50 mg L^–1^ NOM, and 10 mM CaCl_2_ over 10 min of mixing time. The results show the mean ± standard deviation. The calculated (*Ct*_0_/*Ct*) and the natural logarithm of (*Ct*_0_/*Ct*) for the first five data points are shown in the smaller graphs.

We measured the heteroaggregation of PS and PVC with SiO_2_ in the presence of the NOM and CalC_2_. Accordingly, the number of nanoplastics in the dispersion of NOM (50 mg L^–1^), SiO_2_ (20 mg L^–1^), and CaCl_2_ (10 mM) was measured over 10 min (Figure 3). The results showed that the presence of NOM minimized the observed decreases in the numbers of both nanoplastics (PS and PVC) in the dispersion, even in the presence of 100 mM CaCl_2_. This suggests that NOM can stabilize SiO_2_ and subsequently decrease the heteroaggregation between them and the nanoplastics, similar to what has been reported for metallic nanoparticles.^17^ Alimi et al.^26^ reported that, in the absence of NOM, the CCC of PS nanoplastics in CaCl_2_ was independent of particle size. The addition of humic acid enhanced aggregation via bridging, regardless of the size of the plastics, while fulvic acid had little to no effect. In the NOM, alginate stabilized the particle suspensions. The composition of the used NOM in this study was described previously.^32^ The adsorption of NOM to the surface of nanoplastics and SiO_2_ can lead to steric repulsion between the particles, where the NOM molecules form a protective layer that prevents close interaction and aggregation.^33^ This phenomenon is significant in aquatic environments, where NOM concentrations and qualities vary widely and influence the dispersion and stability of nanoplastics.

### Calculated Attachment Efficiency for Different Water Conditions

We calculated the α from the particle removal results using eq 2. The results (Table 1) showed that α for PS–SiO_2_ was slightly higher than that for PVC–SiO_2_. Indeed, the observed differences in α between PS–SiO_2_ and PVC–SiO_2_ despite identical size and shape of PS and PVC nanoplastics likely stem from variations in other physicochemical properties, e.g., density and hydrophobicity. Differences in density affect the buoyancy and settling rates of nanoplastics in a suspension. Higher-density particles may settle more quickly, influencing the observed aggregation rates. Additionally, denser particles might interact differently with other particles or with the surrounding medium, affecting the aggregation behavior. More hydrophobic particles are likely to aggregate more readily due to stronger van der Waals forces and reduced repulsion between particles. Hydrophobic interactions can lead to increased clustering and aggregation, especially in nonaqueous environments or in the presence of hydrophobic substances. These variations can influence the surface properties, and interaction potentials of the tested nanoparticles with SiO_2_, thereby impacting their α under changing ionic strength conditions. However, it was observed that α values of nanoplastics and SiO_2_ decreased with increasing concentrations of CaCl_2_ for both PS and PVC particles. This outcome was anticipated as an elevation in ionic strength (i.e., CaCl_2_) is known to contract the electrical double layer surrounding the particles, thereby promoting heteroaggregation between nanoplastics and SiO_2_ because of the attraction generated by van der Waals forces.^8,34^

**Table 1:** Calculated α for Nanoplastics of Different Chemical Compositions and SiO_2_ under Different Water Conditions^a^

nanoplastics-SiO_2_	water condition: pH: 8, MQ water	α_global_
PS–SiO_2_	0.1 mM CaCl_2_	0.24 ± 0.03
	1 mM CaCl_2_	0.31 ± 0.051
	10 mM CaCl_2_	0.37 ± 0.05
	50 mM CaCl_2_	0.6 ± 0.08
	50 mg L^–1^ NOM, 10 mM CaCl_2_	0.03 ± 0.001
PVC–SiO_2_	0.1 mM CaCl_2_	0.34 ± 0.04
	1 mM CaCl_2_	0.5 ± 0.07
	10 mM CaCl_2_	0.5 ± 0.06
	50 mM CaCl_2_	0.8 ± 0.07
	50 mg L^–1^ NOM, 10 mM CaCl_2_	0.05 ± 0.001

a NOM: natural organic matter.

The introduction of 50 mg L^–1^ NOM resulted in a decrease in α even in the presence of 10 mM CaCl_2_. NOM, a complex mixture of organic compounds derived from the degradation of plant and animal materials such as humic and fulvic acids, can attach to the surface of SiO_2_ and nanoplastics.^8^ The presence of NOM on particle surfaces can induce repulsion among the particles.^33^ Consequently, this repulsion effect diminishes the likelihood of particle attachment, thereby reducing α and minimizing the formation of heteroaggregation.

### Fate Model Output Analysis

**Figure 4:**
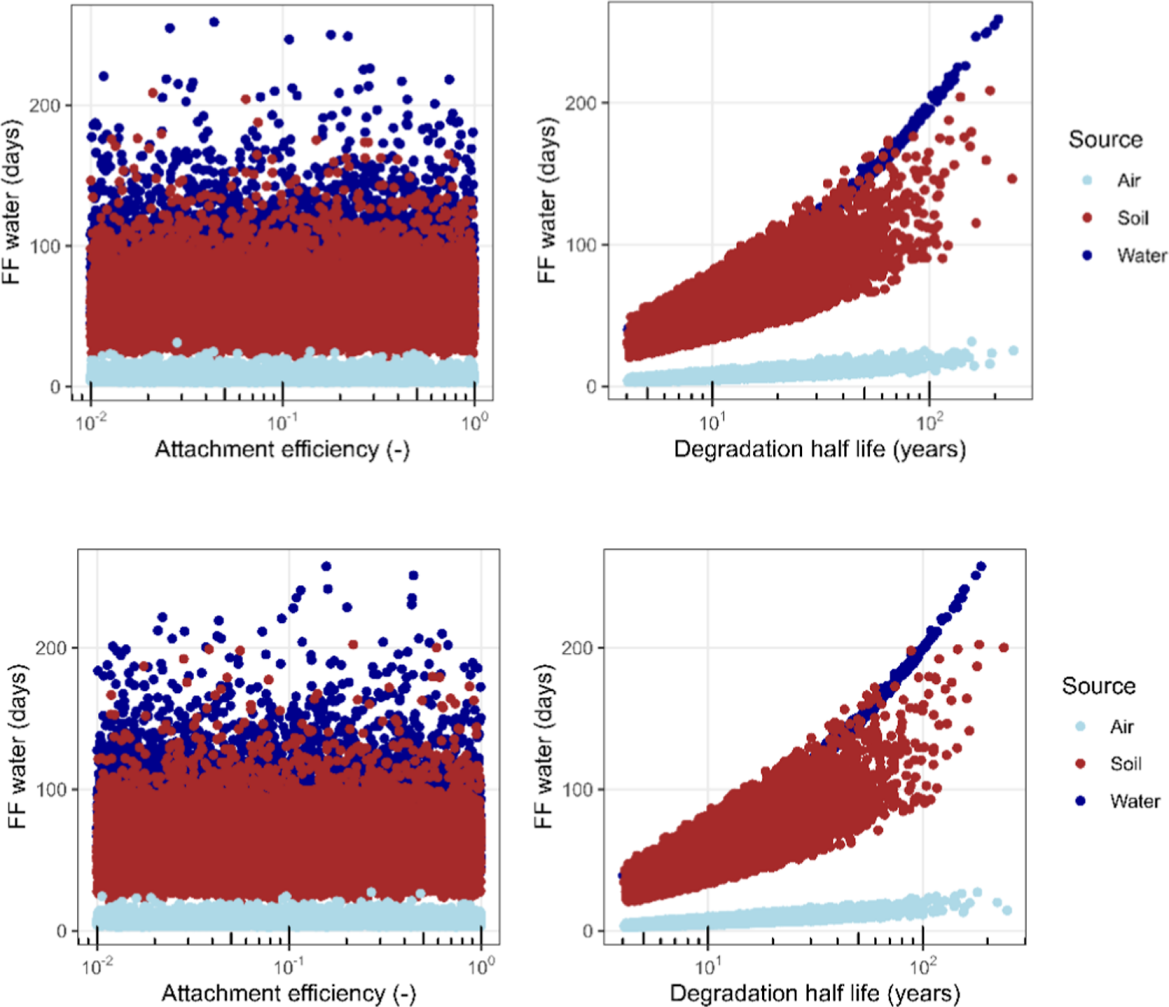
Fate factors for freshwater versus variability in attachment efficiency and soil/sediment degradation half-life for PVC (top) and PS (bottom). The rest of the figures are provided in the Supporting Information, Figures S3–S8.

Uncertainties in particle characteristics, such as size, density, attachment efficiency, and degradation half-life, were incorporated into the SB4P model. This study marks the first exact measurements of α for nanoplastics. Surprisingly, the results indicate that variations in α, especially in scenarios with and without dissolved NOM, do not significantly affect FF. It is assumed that natural waters typically contain some level of often very heterogeneous NOM, suggesting that attachment efficiencies likely range between 0.01 and 1 depending on the NOM concentration and type as well as water salinity. Despite these ranges, no substantial impact on the FF was observed, as shown in Figure 4.

However, the degradation half-life of PS and PVC emerged as a more critical factor, significantly influencing the persistence of PVC and PS in the environment. Although these were not measured for PVC and PS specifically, roughly 3 orders of magnitude range are taken, based on earlier estimates,^14^ compared to only two for the α. Nevertheless, a clear relationship was seen, with almost all of the variability for FF in soil and sediment related to the degradation half-life in those compartments. This is in line with an earlier environmental fate modeling study on metallic nanoparticles^12^ where the predicted environmental concentration was sensitive to degradation half-life, which was larger than 0.5 years, and attachment efficiencies lower than 10^–4^. It remains to be determined whether other environmental factors, such as pH, could further reduce α to such low values. The data presented suggest that in the presence of NOM, the α decreases by about 1 order of magnitude. It is important to note that the model scenario does not account for all local variability in terrestrial and aquatic systems. Additionally, using SiO_2_ as a model simplifies the complexity of natural suspended particulate matter (SPM) and other colloids present in the environment. However, given the ubiquity of SiO_2_ particles, the concentration and number of particles per unit surface area of SPM and natural colloids are likely to influence heteroagglomeration more significantly than the type of particle itself.

Furthermore, the calculated FF indicates that the highest values are observed for PVC and PS ending up in soil and freshwater sediment compartments (Table 2). The emission route significantly influences these outcomes; emissions to soil result in the majority of PVC and PS being retained in the soil compartment, whereas emissions to water lead to accumulation primarily in freshwater sediment. Specifically, for both PS and PVC particles emitted to air, significant amounts are found in seawater, followed by soil and air compartments. When emitted to the soil, both PS and PVC exhibit substantial retention in soil and freshwater sediment compartments regardless of emission pathways. Conversely, particles emitted directly into water predominantly accumulate in freshwater sediment. These findings underscore the importance of emission pathways in determining the environmental distribution and fate of nanoplastics like PS and PVC, highlighting potential ecological implications across different environmental compartments.

**Table 2:** Average Fate Factors (FF) Calculated per Source Route and Environmental Compartment^a^

source	nanoplastic	N	air	freshwater sediment	freshwater	marine sediment	seawater	soil
air	PS	10,000	5.30	1.24	7.10	0.23	36.09	8.56
air	PVC	10,000	5.25	1.58	7.47	0.26	35.99	9.19
soil	PS	10,000	na	10.09	58.21	0.23	46.36	123.42
soil	PVC	10,000	na	12.15	57.61	0.28	45.70	122.70
water	PS	10,000	na	105.08	76.76	1.14	54.28	na
water	PVC	10,000	na	106.53	76.10	1.18	53.59	na

a na: not available, indicating model output is 0 kg in these compartments resulting in a FF of 0.

Our study provides critical insights into the aggregation kinetics and environmental fate of PS and PVC nanoplastics. The experimental findings demonstrated that nanoplastics are highly stable against homoaggregation but prone to heteroaggregation with SiO_2_ under high ionic strength. The presence of NOM significantly reduces the level of aggregation, highlighting its role in stabilizing nanoplastics in natural waters. Importantly, our fate modeling showed that degradation half-life, rather than attachment efficiency, is the primary factor influencing the persistence of nanoplastics in the environment, particularly in soil and freshwater sediments. These findings underscore the necessity of considering both chemical and environmental factors such as NOM and ionic strength when predicting the fate of nanoplastics. This research advances the understanding of nanoplastic behavior and highlights the need for further investigation into their long-term ecological impact across different environmental compartments.

The high stability of PS and PVC nanoplastics against homoaggregation suggests prolonged dispersion in natural waters, potentially increasing their bioavailability to aquatic organisms. The observed heteroaggregation with SiO_2_ under high ionic strength conditions indicates that nanoplastics may settle into sediments of marine environments, posing long-term risks to benthic ecosystems. Furthermore, the reduction in aggregation due to NOM suggests that nanoplastics may remain suspended for longer periods in water bodies rich in organic material, enhancing their transport over long distances and increasing the risk of incorporation by organisms such as plants and animals, and thus the health risk for humans.

The fate modeling results highlight that nanoplastics emitted into air, freshwater, or soil predominantly accumulate in soil and sediment compartments, raising concerns about their persistence in terrestrial and aquatic environments. The significant influence of the degradation half-life on the fate of these particles underscores the need for accurate degradation rates in environmental risk assessments. These findings stress the potential for nanoplastics to act as long-term environmental contaminants, necessitating the development of targeted mitigation strategies to reduce their release and enhance their degradation in natural ecosystems. The role of emission pathways in determining their environmental distribution also suggests that regulations should focus on minimizing plastic emissions, particularly in regions with vulnerable ecosystems.

## Supporting Information Available

spICP-MS settings, including radio frequency power, gas flows, dwell time, and acquisition time, ensuring precise nanoplastic characterization; EUSES settings in SB4P used for model parameterization to simulate the fate and transport of PS and PVC nanoplastics; experimentally determined substance properties, such as particle radii, density, and Hamaker constants incorporated into the model using triangular or uniform distributions; attachment efficiencies and degradation rates parameterized using data from experiments under varying environmental conditions; and detailed overview of the parameters, distributions, and fate factor calculations supporting the analysis (PDF)
