# Supplementary material for: Importance of Attachment Efficiency in Determining the Fate of PS and PVC Nanoplastic Heteroaggregation with Natural Colloids Using a Multimedia Model
Source: Environ Sci Technol. 2025 Mar 3;59(9):4674–83. doi: 10.1021/acs.est.4c10918 (PMC11912305; doi:10.1021/acs.est.4c10918)
Supplement: Supplementary file 1 — es4c10918_si_001.pdf [file es4c10918_si_001.pdf]

Supporting Information

The importance of attachment efficiency in determining the fate of PS and PVC nanoplastics heteroaggregation with natural colloids using a multimedia model

Fazel Abdolahpur Monikh[1,2,3*], Joris T.K. Quik[4], Mark R. Wiesner[5,6], Andrea Tapparo[1], Paolo Pastore[1], Hans-Peter Grossart[7,8], Jarkko Akkanen[2], Raine Kortet[2], Jussi Kukkonen[2,9]

[1] Department of Chemical Sciences, University of Padua, Via Francesco Marzolo, 1, 35122, Padua, Italy.
[2] Department of Environmental and Biological Sciences, University of Eastern Finland, Joensuu 80101, Finland,
[3] Institute for Nanomaterials, Advanced Technologies, and Innovation, Technical University of Liberec Bendlova 1409/7, 460 01, Liberec, Czech Republic
[4] National Institute for Public Health and Environment (RIVM), Centre for Sustainability, Health and Environment, Antonie van Leeuwenhoeklaan 9, 3721 MA Bilthoven, The Netherlands
[5] Department of Civil and Environmental Engineering, Duke University, Durham, North Carolina 27708, United States
[6] Center for the Environmental Implications of NanoTechnology (CEINT), Duke University, Durham, North Carolina 27708, United States
[7] Department of Plankton and Microbial Ecology, Leibniz Institute for Freshwater Ecology and Inland Fisheries, 16775 Stechlin, Germany; Institute of
[8] Biochemistry and Biology, Potsdam University, 14469, Potsdam, Germany
[9] Department of Environmental and Biological Sciences, University of Eastern Finland, Kuopio 70211, Finland.

* *Corresponding Author*
Email: Fazel.Monikh@unipd.it
ORCID: Fazel A. Monikh 0000-0001-9500-5303

Summary: 9 pages, 8 figures, 7 table.

Section 1: ICP-MS

The setup of the spICP-MS measurement Table S1.

Table S1. Single-particle inductively coupled plasma mass spectrometry (sPICP-MS) settings

**Single-particle ICP-MS parameters**	
Radio frequency power	1600 W
Nebulizer type	Quartz nebulizer for NexIONs 2000
Spray chamber type	Glass cyclonic
Plasma gas flow	18 L/min
Nebulizer gas flow	1.2 L/min
Auxiliary gas flow	1.12 L/min
Dwell time	80 µs
Acquisition time	100 s

Section 2: Model parametrization

Settings

Under Substance, for ChemName, 'microplastic' is selected. This refers to several properties that will be partly overwritten by our own selected and generated data. ScenarioName is set to 'EUSES settings' since these are especially fit for modelling at the European scale. For this study, the scenario settings only matter for the fraction of compartments. CaseName is 'defaults only' because we have no reason to differentiate from the defaults.

Substance properties

The experiments were performed on PS with a diameter of 270mm (SD=13) and on PVC with a diameter of 288mm (SD=30) (Fazel, personal communication). The model requires this to be converted to radii. A triangle distribution will be used with a minimum and maximum of 2 SD, as listed in Table S1, to account for differences in the particles. For density [kg m^{-3}], a uniform distribution is used using the values from different sources as minimum or maximum. The Hamaker constant for both substances is calculated through the following formula:[1]

$$(2)\ A_{ENP,\,water,\,NCP} = \left(\sqrt{A_{ENP}} - \sqrt{A_{water}}\right)\left(\sqrt{A_{NCP}} - \sqrt{A_{water}}\right)$$

[Eq. S1]

Awater: https://webspace.clarkson.edu/projects/crcd/public_html/me437/downloads/5_vanderWaals.pdf [1]

A_ENP: PVC=8.17*10^-20

PS=6.88*10^-20

ASiO2: 6.5*10^-20
https://www.eng.uc.edu/~beaucag/Classes/Properties%20of%20Materials/HamakerConstPaper.pdf

Table S2. Link between 3 emission compartments and calculation of 17 Fate Factors

Emission compartment	Resulting FF for
Air	Air, Freshwater, Freshwater Sediment, Soil, Sea water, Marine Sediment
Water	Freshwater, Freshwater Sediment, Sea water, Marine Sediment
Soil	Freshwater, Freshwater Sediment, Soil, Sea water, Marine Sediment

Section 3: Particle characterization

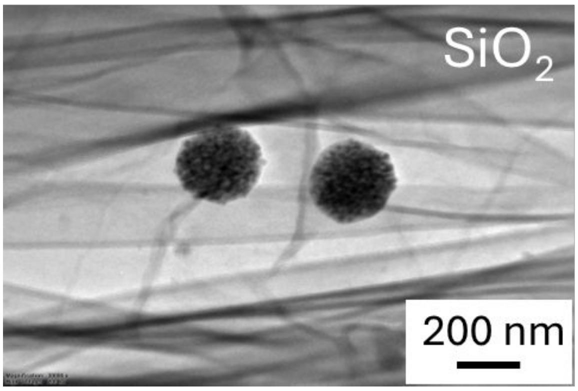

Figure S1. TEM image of SiO_2 particles.

Number of nanoparticles measured using different instruments

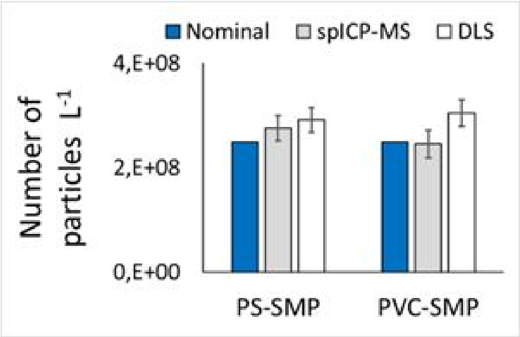

Figure S2. To ensure that most of the nanoplastics particles have a measurable amount of Gd, we compared the nominal number of particles with the number of particles measured by single particle Inductively coupled plasma mass spectrometry (spICP-MS) and dynamic light scattering (DLS).

Table. S3. Zeta potential of PS and PVC in different Ca solution

**Nanoplastics**	**Ca concentration mM**				
	**10 mM**	**50 mM**	**100 mM**	**500 mM**	**1000 mM**
PS	-44 ± 2 mV	-40 ± 3 mV	-36 ± 2 mV	-20 ± 4 mV	-15 ± 2 mV
PVC	-46 ± 1 mV	-43 ± 3 mV	-40 ± 4 mV	-22 ± 3 mV	-17 ± 4 mV
SiO_2	-23 ± 3 mV	-13 ± 2 mV	-5 ± 4 mV	-2 ± 4 mV	10 ± 3 mV

Section 5: Detailed results for Fate Factor calculations for PVC and PS nanoplastics

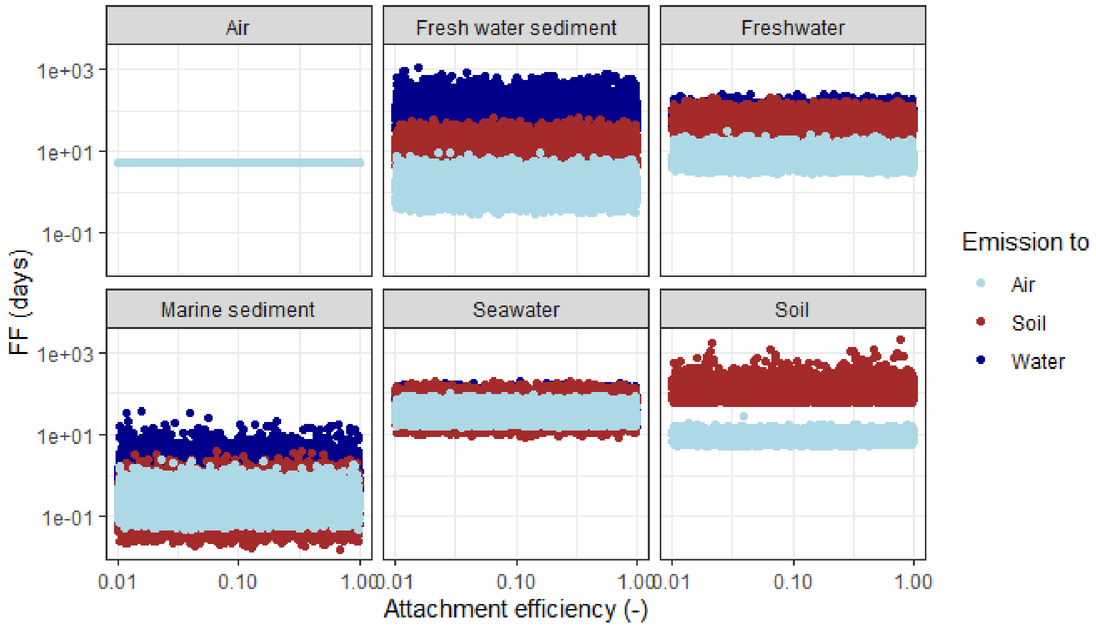

Figure S3. Fate Factors for PVC versus attachment efficiency as calculated using SimpleBox4Plastics (n=10000) at Continental scale (resembling EU) for different compartments (soil and freshwater sub-compartments aggregated) for emission routes through air, soil and water.

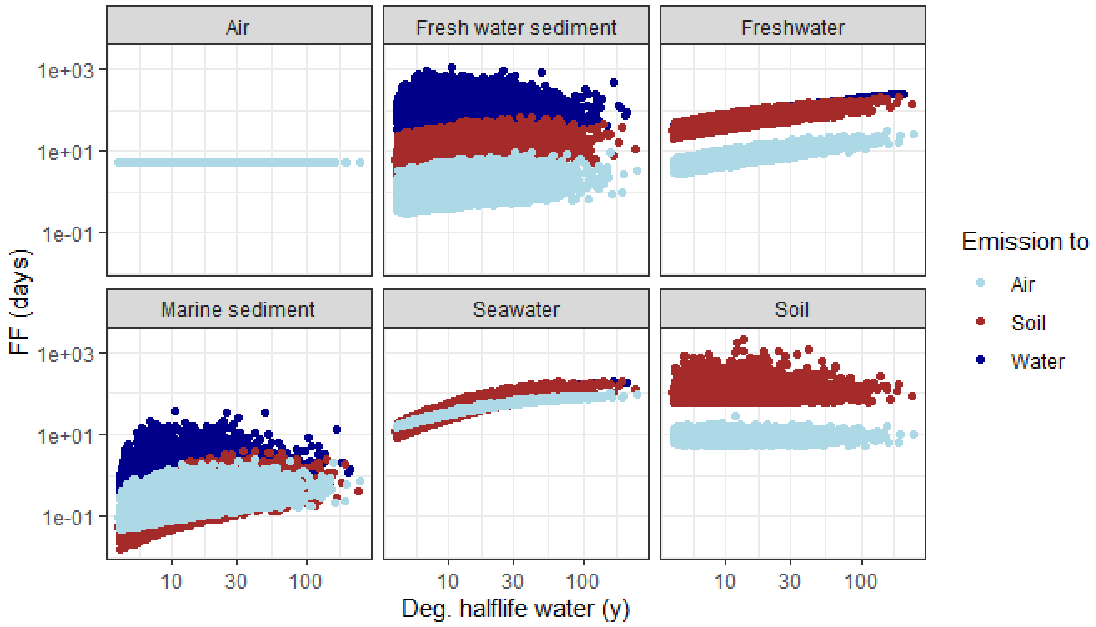

Figure S4. Fate Factors for PVC versus water degradation half-life as calculated using SimpleBox4Plastics (n=10000) at Continental scale (resembling EU) for different compartments (soil and freshwater sub-compartments aggregated) for emission routes through air, soil and water.

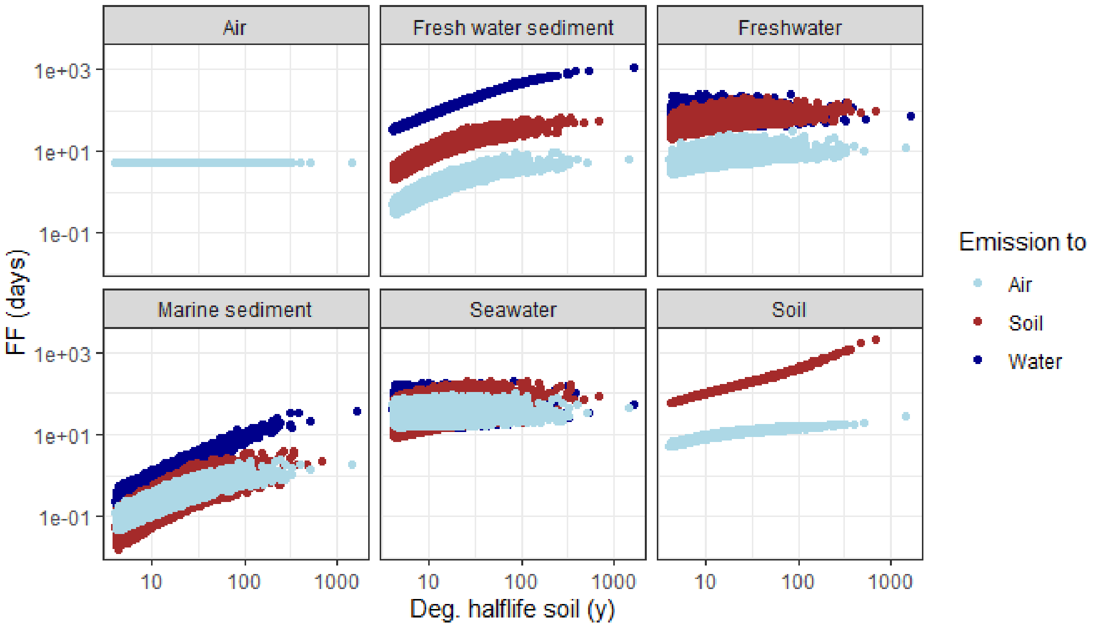

Figure S5. Fate Factors for PVC versus soil degradation half-life as calculated using SimpleBox4Plastics (n=10000) at Continental scale (resembling EU) for different compartments (soil and freshwater sub-compartments aggregated) for emission routes through air, soil and water.

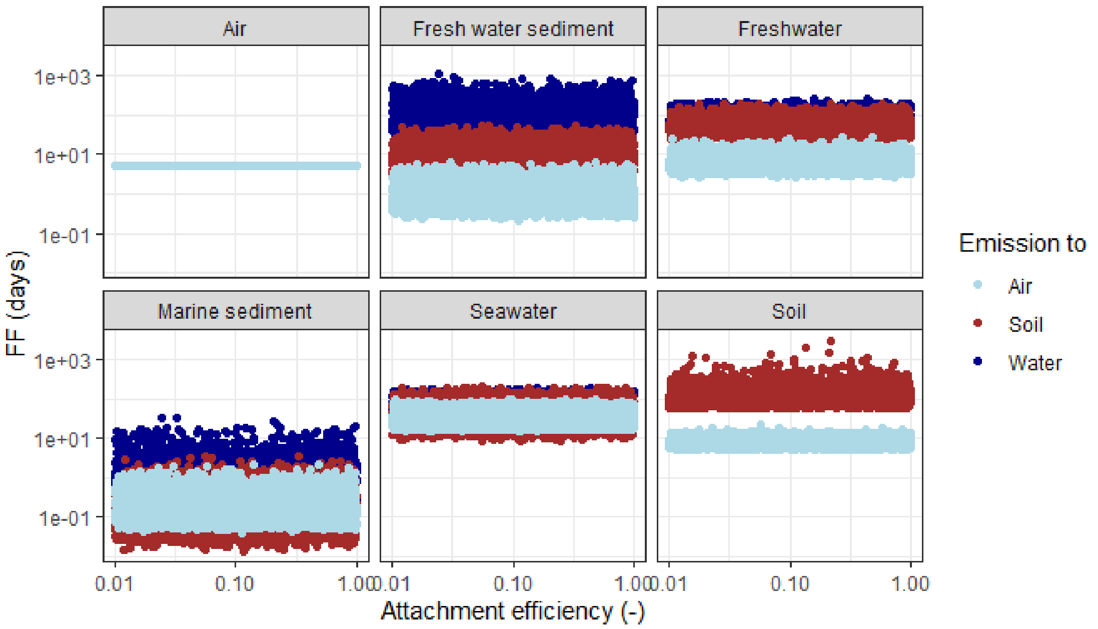

Figure S6. Fate Factors for PS versus attachment efficiency as calculated using SimpleBox4Plastics (n=10000) at Continental scale (resembling EU) for different compartments (soil and freshwater sub-compartments aggregated) for emission routes through air, soil and water.

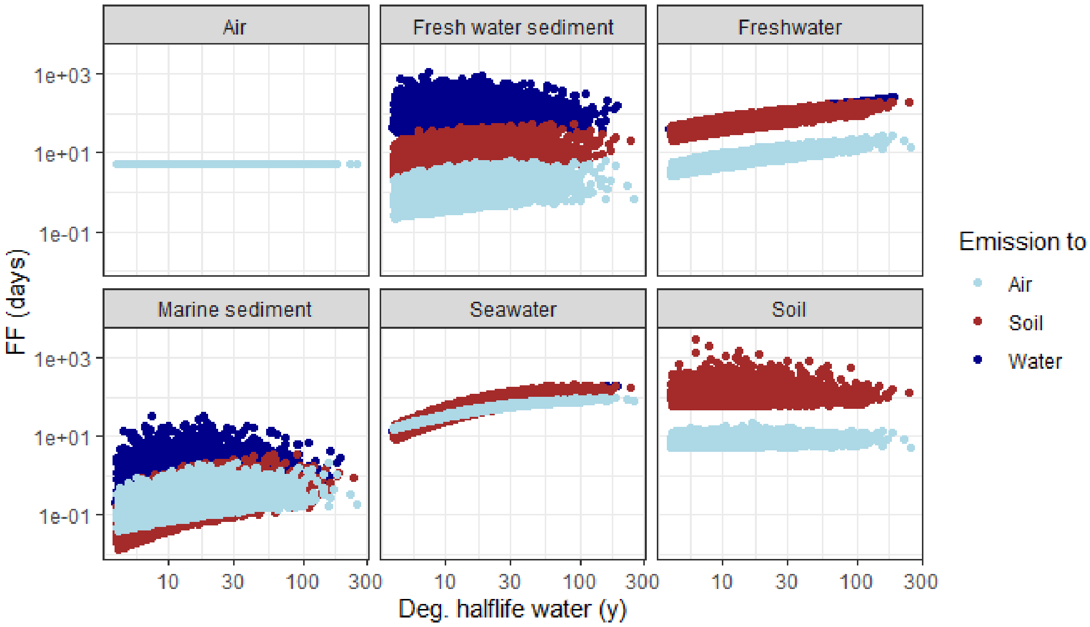

Figure S7. Fate Factors for PS versus water degradation half-life as calculated using SimpleBox4Plastics (n=10000) at Continental scale (resembling EU) for different compartments (soil and freshwater sub-compartments aggregated) for emission routes through air, soil and water.

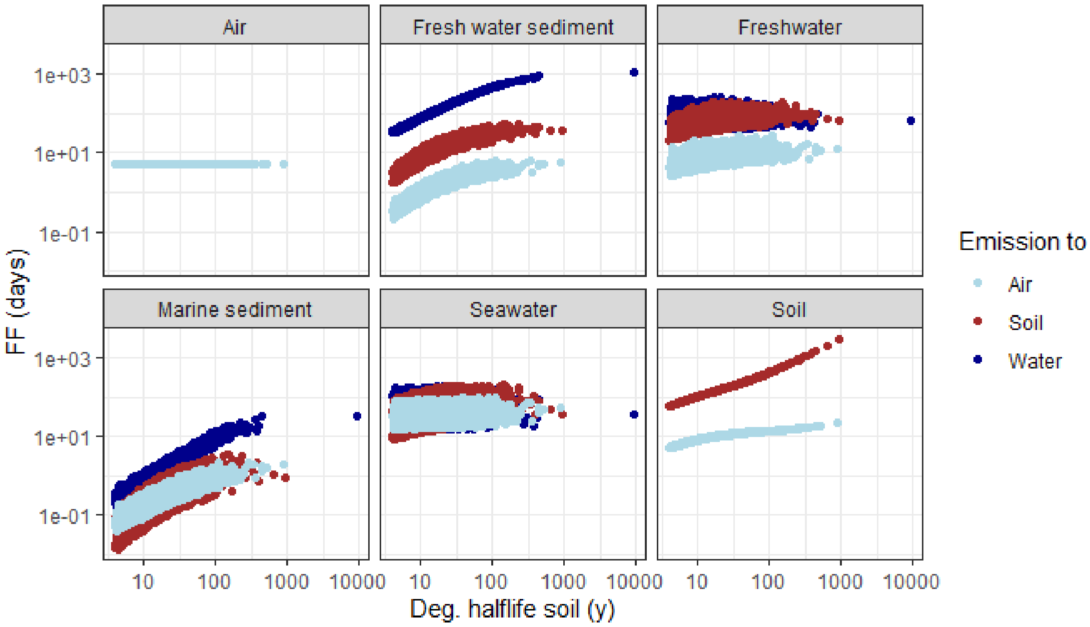

Figure S8. Fate Factors for PS versus soil degradation half-life as calculated using SimpleBox4Plastics (n=10000) at Continental scale (resembling EU) for different compartments (soil and freshwater sub-compartments aggregated) for emission routes through air, soil and water.

Table S4. Substance properties used in SB4P.

Parameters	Material	Minimum value	Most likely value	Maximum value	Type of distribution	Source
Radius [mm]	PS	122	135	148	Triangle	Data
Radius [mm]	PVC	114	144	174	Triangle	Data
Density [kg m^{-3}]	PS	1000		1200	Uniform	Scipoly et al.
Density [kg m^{-3}]	PVC	1380		1400	Uniform	Scipoly et al.
Hamaker constant [J]	PS		5.85E-21		Constant	Calculated
Hamaker constant [J]	PVC		4.39E-21		Constant	Calculated
Attachment Efficiency	PS & PVC	0.01		1	Uniform	Covering a range of data, see also Table S3
Degradation – removal water	PS & PVC	1E-20	1E-10	2E-09	Triangle	Based on Chamas 2021, see also table S4
Degradation – removal soil/sediment	PS & PVC	1E-20	3E-11	1E-9	Triangle	Based on Chamas 2021, see also table S4
Fragmentation – removal water	PS & PVC	1.6E-9	2.7E-8	1.3E-7	Triangle	Based on Koelmans 2017 and Kaandrop 2021, see also table S4
Fragmentation – removal soil/sediment	PS & PVC	1E-20	2.7E-8	1.3E7	Triangle	Based on Koelmans 2017 and Kaandrop 2021, see also table S4

Attachment efficiencies

The attachment efficiency was tested five times for PS and PVC under different concentrations of $CaCl_2$ and NOM. These represent a wide variety of possible environmental conditions. Preferably, different attached efficiencies for different compartments are used because it is known that they differ. However, it is not possible to link some water conditions from the experiment to the different media in SB4P. Therefore, the five experiments are taken together, and the lowest and highest outcomes are used for a uniform distribution in SB4P. Since these outcomes differ more than an order of magnitude, it is better to apply @Risk to a logarithmic distribution as is shown in Table S5. These values are transformed back by using them as an exponent to 10.

Table S5. Attachment efficiencies used in SB4P.

	Minimum value from experiment	Maximum value from experiment	Min-log10	Max-log10	Range in SB4P
PS	0.03	0.6	-2	0	0.01-1
PVC	0.05	0.8	-2	0	0.01-1

Dissolution rates

Dissolution rates are assumed to be zero, similar to e.g. [2], considering the properties of microplastics.

Degradation and transformation

The degradation and transformation rates are assumed to be similar to those in [2] see Table 3. The minimum and maximum values are used in an uniform distribution. A transformation to and back transformation from logarithmic value is used in the same way as explained for the attachment efficiency.

Table S6. Degradation and transformations rates for SB4P.

Rate constant	Source	Min (s^{-1})	Average (s^{-1})	Max (s^{-1})	Method of extrapolation
$k_{frag\text{-}air}$	-	-	0	-	
$k_{mpdeg\text{-}air}$	-	-	0	-	
$k_{frag\text{-}water}$ (applied to sea (w2) and fresh water (w0,w1))	Koelmans 2017 and Kaandorp 2021	1.6E-9	2.7E-8	1.3E-7	Max based on an estimate from[3]. Assumed no fragmentation in deep seawater.
$K_{mpdeg\text{-}water}$ (applied to surface (w2) and deep sea (w3) and fresh water (w0, w1))	Chamas 2021	1e-20	1E-10	2E-9	
$K_{frag\text{-}soil}$ (applied to all) & $K_{frag\text{-}sediment}$ (applied to all)	Koelmans 2017 and Kaandorp 2021	1e-20	2.7E-8	1.3E-7	As soil can be less dynamic in terms of mixing and shear compared to surface seawater and the lack of information (large uncertainty) the minimum fragmentation rate constant is set to near-zero: 1e-20 s^{-1}
$K_{mpdeg\text{-}soil}$ (all) $K_{mpdeg\text{-}sediment}$ (all)	Chamas 2021	1e-20	3E-11	1E-9	

Emission

One of the three possible emission routes is set to a hypothetical 1 t yr^{-1}. Considering the calculations for fate factors, the exact value is irrelevant. Because SB4P asks for seven emissions, the emission to each of these compartments needs to be corrected for the fraction of the total area. So three compartments of both water and soil both add up to 1. The emissions to either air, water and soil are run separately to be able to differentiate the output (Table S4).

Table S7. Emission values for SB4P

Compartments reported in this study	Air	Water			Soil		
Compartments in SB4P	Air	Lake water	Fresh water		Natural soil	Agricultural soil	Other soil
kg/day	1	0.08	0.92		0.28	0.62	0.1

Section 4: Determining the α

The modified Smoluchowski equation (SI Eq. 1) delineates the transport processes guiding a particles towards the surface of another particles, and the likelihood of attachment ensuing from a collision between these particles. It is formulated through the multiplication of several factors: the dimensionless attachment efficiency (α), the second-order collision rate constant (β) measured in volume $time^{-1}$ $number^{-1}$, and the concentrations of colloid particles (B) and nanoparticles (n) measured in number $volume^{-1}$.

$$\frac{dn}{dt} = -\alpha\beta\,(B,n)Bn$$

References:

(1) Wang, C. qing; Wang, H.; Gu, G. hua; Fu, J. gang; Lin, Q. quan; Liu, Y. nian. Interfacial Interactions between Plastic Particles in Plastics Flotation. *Waste Manag.* **2015**, *46*, 56–61. https://doi.org/10.1016/j.wasman.2015.08.041.

(2) Quik, J. T. K.; Meesters, J. A. J.; Koelmans, A. A. A Multimedia Model to Estimate the Environmental Fate of Microplastic Particles. *Sci. Total Environ.* **2023**, *882*, 163437. https://doi.org/10.1016/j.scitotenv.2023.163437.

(3) Meesters, J. A. J.; Peijnenburg, W. J. G. M.; Hendriks, A. J.; Van De Meent, D.; Quik, J. T. K. A Model Sensitivity Analysis to Determine the Most Important Physicochemical Properties Driving Environmental Fate and Exposure of Engineered Nanoparticles. *Environ. Sci. Nano* **2019**, *6* (7), 2049–2060. https://doi.org/10.1039/c9en00117d.
